# Automated Analysis of Proliferating Cells Spatial Organisation Predicts Prognosis in Lung Neuroendocrine Neoplasms

**DOI:** 10.3390/cancers13194875

**Published:** 2021-09-29

**Authors:** Matteo Bulloni, Giada Sandrini, Irene Stacchiotti, Massimo Barberis, Fiorella Calabrese, Lina Carvalho, Gabriella Fontanini, Greta Alì, Francesco Fortarezza, Paul Hofman, Veronique Hofman, Izidor Kern, Eugenio Maiorano, Roberta Maragliano, Deborah Marchiori, Jasna Metovic, Mauro Papotti, Federica Pezzuto, Eleonora Pisa, Myriam Remmelink, Gabriella Serio, Andrea Marzullo, Senia Maria Rosaria Trabucco, Antonio Pennella, Angela De Palma, Giuseppe Marulli, Ambrogio Fassina, Valeria Maffeis, Gabriella Nesi, Salma Naheed, Federico Rea, Christian H. Ottensmeier, Fausto Sessa, Silvia Uccella, Giuseppe Pelosi, Linda Pattini

**Affiliations:** 1 Department of Electronics, Information and Bioengineering, Politecnico di Milano, 20133 Milan, Italy; matteo.bulloni@polimi.it (M.B.); giada.sandrini@mail.polimi.it (G.S.); irene.stacchiotti@mail.polimi.it (I.S.); 2 Division of Pathology, IRCCS European Institute of Oncology, 20136 Milan, Italy; massimo.barberis@ieo.it (M.B.); Eleonora.Pisa@ieo.it (E.P.); 3 Pathology Unit, Department of Cardiac, Thoracic, Vascular Sciences, and Public Health, Medical School, University of Padua, 35122 Padua, Italy; fiorella.calabrese@unipd.it (F.C.); federica.pezzuto@unipd.it (F.P.); 4 Anatomical Pathology Unit-Hospitais da Universidade de Coimbra/Centro Hospitalar e Universitário de Coimbra-Portugal, Faculty of Medicine, University of Coimbra-Portugal, 3004-504 Coimbra, Portugal; lcarvalho@chuc.min-saude.pt; 5 Department of Surgical Pathology, Medical, Molecular and Critical Area, University of Pisa, 56126 Pisa, Italy; gabriella.fontanini@med.unipi.it; 6 Operative Unit of Anatomic Pathology, Azienda Ospedaliero-Universitaria Pisana, 56126 Pisa, Italy; greta.ali@gmail.com (G.A.); francesco.fortarezza@unipd.it (F.F.); 7 Laboratory of Clinical and Experimental Pathology, FHU OncoAge, Louis Pasteur Hospital BB-0033-00025, IRCAN, Université Côte d’Azur, 06100 Nice, France; hofman.p@chu-nice.fr (P.H.); Hofman.v@chu-nice.fr (V.H.); 8 Department of Pathology, University Clinic of Respiratory and Allergic Diseases Golnik, 4204 Golnik, Slovenia; Izidor.Kern@klinika-golnik.si; 9 Pathology Section, Department of Emergency and Organ Transplantation, University of Bari, 70121 Bari, Italy; eugenio.maiorano@uniba.it (E.M.); gabriella.serio1@uniba.it (G.S.); andrea.marzullo@uniba.it (A.M.); seniatrabucco@hotmail.com (S.M.R.T.); 10 Pathology Unit, Department of Medicine and Surgery, University of Insubria, 21100 Varese, Italy; roberta.maragliano@gmail.com (R.M.); marchiori.deborah93@gmail.com (D.M.); fausto.sessa@uninsubria.it (F.S.); silvia.uccella@uninsubria.it (S.U.); 11 Department of Oncology, University of Turin, 10124 Turin, Italy; jasna.metovic@unito.it (J.M.); mauro.papotti@unito.it (M.P.); 12 Department of Pathology, Erasme Hospital, Université Libre de Bruxelles, 1050 Brussels, Belgium; myriam.remmelink@erasme.ulb.ac.be; 13 Pathology Division, Department of Surgery, University of Foggia, 71122 Foggia, Italy; antonio.pennellla@unifg.it; 14 Thoracic Surgery Section, Department of Surgery and Organ Transplantation, University of Bari, 70121 Bari, Italy; angela.depalma@uniba.it (A.D.P.); giuseppe.marulli@uniba.it (G.M.); 15 Surgical Pathology & Cytopathology Unit, Department of Medicine (DIMED), University of Padova, Via Aristide Gabelli, 61, 35121 Padova, Italy; ambrogio.fassina@unipd.it (A.F.); maffeisv@gmail.com (V.M.); 16 Department of Health Sciences, University of Florence, 50139 Florence, Italy; gabriella.nesi@unifi.it; 17 Cancer Sciences Unit, Faculty of Medicine, University of Southampton, Southampton SO17 1BJ, UK; S.Naheed@soton.ac.uk; 18 Thoracic Surgery Unit, Department of Cardiac, Thoracic, Vascular Sciences, and Public Health, Medical School, University of Padua, 35122 Padua, Italy; federico.rea@unipd.it; 19 Liverpool Head and Neck Centre, Department of Molecular & Clinical Cancer Medicine, Institute of Translational Medicine, University of Liverpool, Liverpool L1 8JX, UK; c.ottensmeier@liverpool.ac.uk; 20 Department of Oncology and Hemato-Oncology, University of Milan, 20122 Milan, Italy; giuseppe.pelosi@unimi.it; 21 Inter-Hospital Pathology Division, IRCCS MultiMedica, 20138 Milan, Italy

**Keywords:** Ki-67, prognosis, lung cancer, lung neuroendocrine neoplasms, histopathology, whole-slide image, machine learning

## Abstract

**Simple Summary:**

Lung neuroendocrine neoplasms (lung NENs) are categorised by morphology, defining a classification sometimes unable to reflect ultimate clinical outcome, particularly for the intermediate domains of adenocarcinomas and large-cell neuroendocrine carcinomas. Moreover, subjectivity and poor reproducibility characterise diagnosis and prognosis assessment of all NENs. The aim of this study was to design and evaluate an objective and reproducible approach to the grading of lung NENs, potentially extendable to other NENs, by exploring a completely new perspective of interpreting the well-recognised proliferation marker Ki-67. We designed an automated pipeline to harvest quantitative information from the spatial distribution of Ki-67-positive cells, analysing its heterogeneity in the entire extent of tumour tissue—which currently represents the main weakness of Ki-67—and employed machine learning techniques to predict prognosis based on this information. Demonstrating the efficacy of the proposed framework would hint at a possible path for the future of grading and classification of NENs.

**Abstract:**

Lung neuroendocrine neoplasms (lung NENs) are categorised by morphology, defining a classification sometimes unable to reflect ultimate clinical outcome. Subjectivity and poor reproducibility characterise diagnosis and prognosis assessment of all NENs. Here, we propose a machine learning framework for tumour prognosis assessment based on a quantitative, automated and repeatable evaluation of the spatial distribution of cells immunohistochemically positive for the proliferation marker Ki-67, performed on the entire extent of high-resolution whole slide images. Combining features from the fields of graph theory, fractality analysis, stochastic geometry and information theory, we describe the topology of replicating cells and predict prognosis in a histology-independent way. We demonstrate how our approach outperforms the well-recognised prognostic role of Ki-67 Labelling Index on a multi-centre dataset comprising the most controversial lung NENs. Moreover, we show that our system identifies arrangement patterns in the cells positive for Ki-67 that appear independently of tumour subtyping. Strikingly, the subset of these features whose presence is also independent of the value of the Labelling Index and the density of Ki-67-positive cells prove to be especially relevant in discerning prognostic classes. These findings disclose a possible path for the future of grading and classification of NENs.

## 1. Introduction

Neuroendocrine neoplasms of the lung (lung NENs) represent about 14% of primary lung neoplasms [1] and 25% of all neuroendocrine tumours [2]. According to 2015 WHO guidelines, they are classified into four histologically defined variants: typical carcinoid (TC), atypical carcinoid (AC), large cell neuroendocrine carcinoma (LCNEC) and small cell carcinoma (SCC) [3]. U.S. incidence statistics report that SCC and TC constitute 86% and 9% of total lung NENs, respectively, while AC and LCNEC together account for the remaining 5%, corresponding to just 9098 cases out of 1.3 million primary lung tumours recorded between 2000 and 2017 [1].

The current classification of lung NENs incorporates a malignancy grading of the tumours, with TC and AC referring to low-grade and intermediate-grade well-differentiated tumours, respectively, and LCNEC and SCC to high-grade, poorly differentiated tumours. In recent years, however, growing evidence has shown how this morphology-based classification and its embodied grading fail to reliably predict prognosis for a substantial fraction of tumours, especially in the intermediate domains of AC and LCNEC [4]. In particular, an increasing number of clues from both clinical and molecular standpoints hint to the existence of a grey area between these two histological categories, with tumours presenting intermediate characteristics: AC-like morphology, LCNEC-like proliferative activity and prognosis and mixed genomic profiles [5,6,7,8,9,10,11,12,13]. These findings suggest a classification of lung NENs independent of histology and based instead on proliferation activity, allowing tumours with similar behaviour and prognosis to be treated accordingly and consistently [4,14,15,16].

Ki-67 antigen and mitotic count represent the consensus proliferation markers for prognosis stratification among neuroendocrine neoplasms (NENs) [16], with Ki-67 recently shown to outperform mitotic count on a large cohort of lung NENs [14]. However, Ki-67, mitotic count and other morphological or immunohistochemical prognostic markers rely on either the simple evaluation of their prevalence on a narrow, subjectively selected tissue region—by counting the percentage of cells positive to the marker in that region—or a merely qualitative inspection, rather than a quantitative assessment [17,18,19,20,21]. These evaluations therefore lack repeatability, and carry inevitable observer bias [19,22,23,24]. Ki-67 is also known for intratumoural heterogeneity of distribution due to the presence of differentially regulated sets of tumour cells [10,23,25,26], particularly in higher grade carcinoids [10]. Due to this heterogeneity, even the role of automated Ki-67-positive (Ki-67^+^) cells detection software has been limited to the computation of simple counting-based descriptors, such as the well-recognised Ki-67 Labelling Index (Ki-67 LI), i.e., the said percentage of Ki-67^+^ cells in a region, and on very narrow regions, as well [22,27,28,29,30,31]. Moreover, spatial heterogeneity has been shown to represent the main cause of discordant Ki-67 LI assessment with respect to manual counting, even when evaluating small areas [25].

All these issues highlight the intrinsic limitations of Ki-67 LI as well as all counting-based proliferation descriptors, which, even if supported by automation, remain error-prone and represent a very limited account of the tumour’s complexion, excessively bounded to the specific evaluated region. Most importantly, such simple descriptors can only carry an extremely limited amount of information, allowing at most a categorical grading based on simple threshold values. A recent WHO expert consensus proposal [16] highlighted the need for computational pathology research on Ki-67, inviting questioning of this traditional concept of categorical grading and opening doors to new, more advanced solutions. Our aim in the present work is, therefore, to explore a completely new perspective of interpreting Ki-67, based on an objective and comprehensive analysis of the topology of stained tumour cells, and to evaluate its ability to predict prognosis in the lung NENs presenting the most challenging outcome assessment: AC and LCNEC.

**Figure 1:**
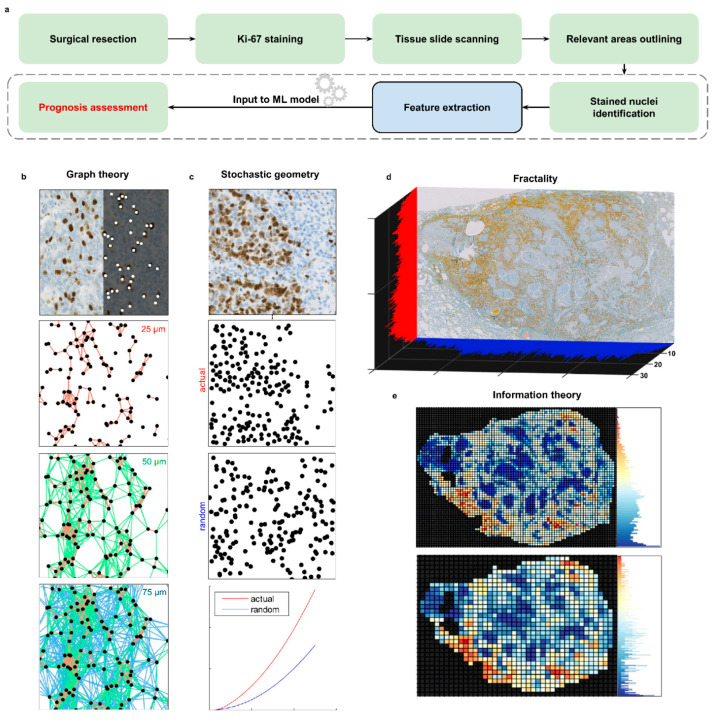
(**a**) Schematic of the steps of our prognosis assessment framework, from input to output. The lower half blocks represent the core procedures carried out by our software implementation. (**b**–**e**) Presentation of the categories of features extracted by our system. (**b**) Graph theory features rely on the construction of graphs from Ki-67+ cells, considering each cell as a node and connecting two nodes if their mutual distance is lower than a fixed proximity threshold. (**c**) Ki-67^+^ cells are interpreted as a stochastic point process; features are derived by measuring how distant the cell spatial arrangement is from a truly random one. (**d**) Visual representation of the preliminary process for evaluating Higuchi’s fractal dimensions of a sample. For each row and column of pixels of the image, the number of Ki-67^+^ cells present on that line is counted, and the existence of fractal patterns is then evaluated on the obtained distributions, shown in red and blue in the figure. The measure presented on the axis is exactly the number of Ki-67^+^ cells found on that pixel row or column. (**e**) Shannon’s entropy of the Ki-67^+^ cell distribution. This measure is evaluated by dividing the image in squared sub-regions, counting the number of replicating cells lying inside each window and evaluating the skewness of the corresponding value distribution. This feature is computed for different sub-region sizes, as exemplified in the panel.

In this study, we present a framework for evaluating proliferation activity and predicting prognosis on the entire extent of high-resolution whole slide images (WSIs), based on an automated and quantitative analysis of the spatial distribution of Ki-67-positive cells and its heterogeneity within the tumour. As shown in Figure 1, our system relies on the integrated evaluation of multiple interpretations of the arrangement of these replicating cells, combining features from the fields of graph theory, fractality analysis, stochastic geometry and information theory to disclose aggressiveness traits undetectable through gold standard counting-based techniques. Requiring just a rough outlining of the neoplastic region of the slide as pre-processing, our system inputs the WSI, identifies Ki-67^+^ cells through an automated detection pipeline, derives quantitative descriptors of their spatial arrangement and, based on this knowledge, predicts overall survival over four years.

## 2. Results

### 2.1. Definition of Prognostic Classes

**Figure 2:**
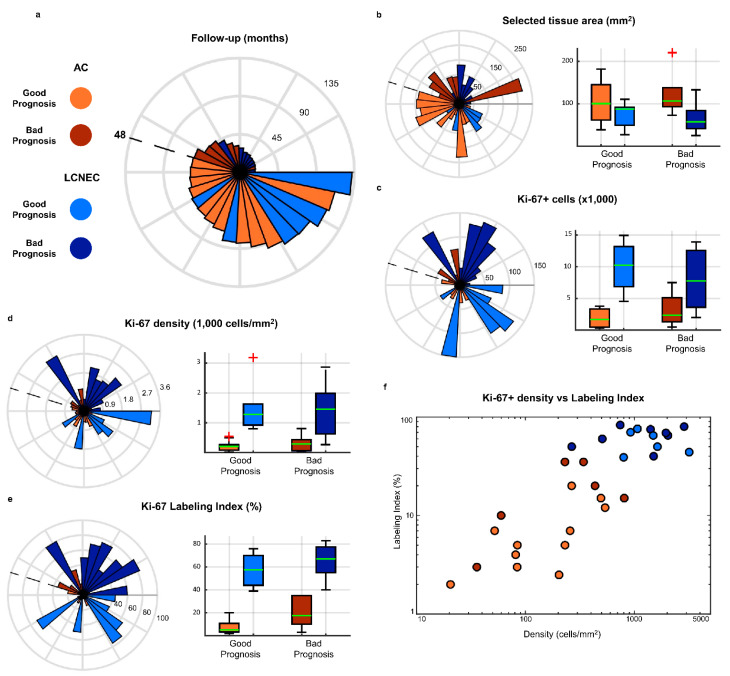
Dataset characteristics. (**a**–**e**) Radial plots of samples’ characteristics, each ordered as in (**a**), to allow comparison; (**b**–**e**) also include the corresponding bar plot, grouped by prognostic class and tumour type. (**a**) Follow-up intervals of patients in months, sorted increasingly counterclockwise. The dashed line represents the separation point between poor prognosis and good prognosis samples, at 48 months. (**b**) Overall outlined area of the WSI after the outlining procedure, i.e., total area on which the identification of Ki-67-positive cells was then performed. (**c**) Number of identified Ki-67^+^ cells. (**d**) Density of Ki-67^+^ cells computed over the entire outlined area. (**e**) Ki-67 Labelling Index, i.e., the manually evaluated percentage of Ki-67^+^ cells over the total number of cells in an area of ≥2 mm^2^, or, equivalently, over at least 2000 cells. (**f**) Relationship between Ki-67^+^ cell’s density and Ki-67 Labelling Index, each presented in logarithmic scale. Correlation analysis between the two yielded *r* = 0.51 and *p* = 0.0032.

Our dataset was composed of 31 whole slide images (WSIs) of surgical resection tissue from as many different patients, collected from medical centres located in Bari, Pisa, Turin and Varese (Italy), Brussels (Belgium) and Nice (France). The dataset included 17 atypical carcinoids (AC) and 14 large cell neuroendocrine carcinomas (LCNEC), with well-spread follow-up intervals ranging from 5 to 131 months. Two prognostic classes were defined: good prognosis (GP) samples—11 AC and six LCNEC—were those from patients presenting no evidence of disease after at least 4 years from surgery, while poor prognosis (PP) samples—six AC and eight LCNEC—belonged to subjects who relapsed and died of disease within 4 years from surgery. A summary of the dataset’s composition and characteristics is presented in Figure 2, while the complete list of images with respective clinical annotations and characteristics is available in Table S1.

### 2.2. Ki-67^+^ Cells Identification

**Figure 3:**
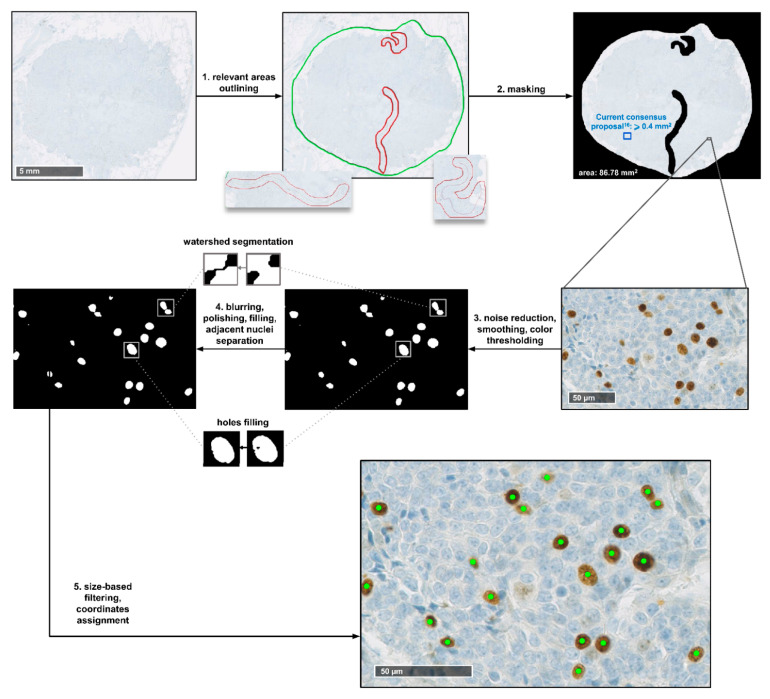
Visual representation of the pipeline of identification of Ki-67^+^ cells in the WSIs.

The first step of our framework consists of the identification of the Ki-67-positive cells on the WSI. The full pipeline of this identification process is shown in Figure 3. The procedure starts with the outlining of the tumour regions of the slide, performed to exclude areas that should not be processed. On NDP.View2 [32], an image viewer for WSIs, utilising just a regular mouse, virtual slide regions containing neoplastic tissue were roughly delimited in green, and any possible area within those regions that presented visual artefacts or necrosis was outlined in red. Any portion of the slide not surrounded by a green line was then discarded, and red-surrounded areas were excluded, as well. Figure 2b shows the distribution of the WSIs in terms of overall outlined area, i.e., the total area available for processing for each image after this step.

After the outlining, the Ki-67^+^ cells identification is performed. Since positivity to anti-Ki-67 antibody for a cell exhibits through brown colouration of the nucleus, the core of this process is the identification of brown-shaded pixels within the image, followed by the recognition of nuclear shapes. The task was carried out through an automated processing pipeline based on colour thresholding and filtering procedures aimed at distinguishing specifically the nuclear shapes within the variegated colour landscape of the image. Figure 2c displays the number of Ki-67^+^ cells identified for each image in our dataset.

### 2.3. Feature Extraction and Selection

The main goal of our work was to verify the possibility of predicting prognosis by deriving and exploiting quantitative descriptors of the spatial distribution of proliferating tumour cells. To achieve this, we investigated multiple possible interpretations of this spatial distribution, relying on essentially different foundations. Four groups of features were derived for each image, corresponding to four different domains of investigation: graph theory, fractality analysis, stochastic geometry and information theory. Graph theory features rely on the interpretation of Ki-67^+^ cell pattern as a network: each positive cell is a node of the network, and nodes close to each other within a certain distance are considered connected. To model contiguity between cells at different scales, we employed three different proximity thresholds, leading to three separate graph models for each image and as many subsets of features. Fractality features, then, are used to inspect the presence of arrangement patterns that appear to occur recurrently in the tumour tissue at different degrees of magnification. In other words, these features capture if the distribution of Ki-67^+^ cells appears to be replicating itself as one progressively “zooms in” on the tissue, and quantify the expression of this behaviour. Stochastic geometry features, instead, assess how distant the entire distribution of Ki-67^+^ cells is from that of a spatial point process, i.e., a pattern of randomly located points in space. The more the cells exhibit some form of spatial aggregation, the higher the magnitude of dissimilarity from a truly random distribution. Lastly, information theory features evaluate how non-random the disposition of Ki-67^+^ cells is by analysing their density within tissue sub-areas. This corresponds to measuring the entropy in the organisation of proliferating cells; the lower the entropy, the higher the chance of a purposeful scheme in their arrangement.

A total of 490 parameters were derived for each image. These include 441 graph theory features, 4 fractality features, 39 stochastic geometry features and 6 information theory features. The definition and computation procedure of each individual feature are described in detail in the Methods section and in the Supplementary Materials Methods S1.

Before setting up the predictive model, to lower the number of parameters and reduce noise, feature selection was carried out. Following a preliminary correlation-based filtering, which discarded 102 of the 490 features, Neighbourhood Component Analysis (NCA) was employed to perform the selection on the remaining ones. NCA ranked the 388 features based on their capability of discerning the prognostic class of the tumour samples, evaluated by means of a multivariate analysis, and assigned a relevance value to each feature. After setting the required relevance threshold to filter the output, NCA yielded a ranking of 23 informative features for our dataset, comprising 21 graph theory features, 1 fractality feature and 1 information theory feature. 

### 2.4. Predictor Evaluation

**Figure 4:**
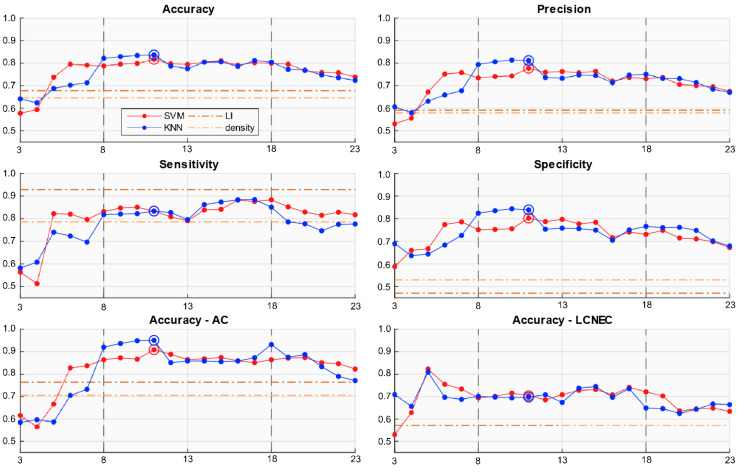
Comparisons of performance metrics between our k-NN and SVM models and the classifiers based on Ki-67 Labelling Index and Ki-67^+^ cell density. The horizontal axes of the plots indicate the number of top prognostically relevant features employed by our models, ranging from top 3 to top 23. The circles are placed in correspondence to the individual models that obtained the best accuracy; for both k-NN and SVM, these correspond to the model that employed 11 features.

To perform the prediction of the prognostic class based on the assessed proliferation activity, we evaluated two machine learning models: k-Nearest Neighbours (k-NN), for k = 3, and Support Vector Machines (SVM). We verified for each model the ability to predict prognostic class employing only the top *n* most relevant features according to feature selection results, with *n* ranging from 3 to 23 (i.e., the total number of meaningful features yielded by NCA). Accuracy, sensitivity, specificity and precision were evaluated for each *n* value, and the results are shown in Figure 4. The two models performed comparably, with k-NN achieving the best accuracy by a slight margin, and SVM showing the best consistency throughout almost the entire range of *n* values. Both models reach a steadily good performance for *n* = 8—SVM slightly earlier—while they struggle for lower *n* values, hinting that the prognosis prediction problem is complex, and its solution requires more information than that carried by a single or even a few parameters. Similarly, the performances of both models appear to decline when *n* > 18. This concurrence suggests that the downturn occurs because the features at the bottom of the relevance ranking are no longer providing useful knowledge, and start introducing noise. Since the exact number of parameters ultimately retained after ranking via NCA was determined by an arbitrary threshold, it is safe to presume that this cut-off value was simply set too low. In light of these considerations, we deem the interval for *n* between 8 and 18 as the meaningful reference for performance. Average metrics for this interval are shown in Figure 4, and reflect the remarkable robustness of performance that both models display throughout the entire span. The fact that two separate models relying on completely different operating principles perform so consistently and reliably as their complexity increases provides strong evidence of how our features truly encode information that is key to discerning the two prognostic classes. Peak performances in terms of classification accuracy were obtained for *n* = 11 for both SVM and k-NN, measuring 81.65% and 83.65%, respectively. For these peak performing models—as well as for all the relevant *n* values—although the first remains slightly higher overall, recall and specificity are comparably close, showing how the system addresses the classification task in a balanced way. This displays how both models effectively learned to recognise the individual characteristics of each prognostic class, rather than overfitting on either of the two. The same holds when considering individual histology accuracies, which, although lower for LCNEC, remain solid and comparable.

### 2.5. Comparison with Ki-67 LI and Ki-67^+^ Cell Density

To evaluate the validity of our results, we performed the same prognostic class prediction basing on the Ki-67 Labelling Index, the currently employed Ki-67 proliferation index for neuroendocrine tumours of the lung and of several other body sites. Additionally, we evaluated as a prediction metric the number of Ki-67^+^ cells per square millimetre computed on the entire WSI. This density value was obtained dividing the number of Ki-67^+^ cells identified within an image by the total area of the WSI on which the identification was performed. Since Ki-67^+^ cells detection on our samples was carried out on the entirety of the tumour tissue available on the image, rather than in specific and narrow areas, we were able to compute and evaluate this additional descriptor. The values of both Ki-67 LI and Ki-67^+^ cell density for each image were shown in Figure 2d,e. For each of these two single-metric classifiers, we found the respective threshold values that maximised classification accuracy—i.e., correctly assigned prognostic class to the highest number of images—and computed the performance measures corresponding to this optimal classification. The optimal threshold value for Ki-67 LI was equal to 10 (i.e., tumours with LI ≥ 10 are classified as poor prognosis cases), while the one for Ki-67^+^ cell density turned out to be 266 cells/mm^2^. The performance of these two optimal classifiers is reported in Figure 4. The LI model predicted the correct prognostic class for 21 out of 31 images, corresponding to an accuracy of 67.74%, while the cell density-based one predicted only 20 (64.52%); both results are far worse than that obtained by our framework, and the same holds for the independent accuracies on AC and LCNEC samples. Moreover, sensitivity and specificity show how the performance was extremely unbalanced on the two prognostic classes.

### 2.6. Feature Analysis

To develop a better understanding of the relationship between our parameters, the two Ki-67 proliferation indices and tumour histology, we performed a series of statistical analyses. These analyses were carried out considering only the 388 features on which NCA was performed, i.e., neglecting those filtered out during the preliminary phase of feature selection, to avoid influencing the results by including plainly redundant parameters. First, we verified the correlation of each feature with Ki-67 LI and Ki-67^+^ cell’s density. A total of 121 features were not correlated in a statistically significant way with the first index, and 111 with the latter. Eighty-seven features, 22.4% of the total 388, were uncorrelated with both indices, showing how Ki-67 LI and Ki-67^+^ cell density are indeed related (as a matter of fact, correlation analysis between the two yielded *r* = 0.51 and *p* = 0.0032). Of these 87, six belong to the 18 features that were the most relevant in discerning prognostic class, with three of them in the top six. Namely, 33.3% of the most prognosis-relevant features, and 50% of the top six, are uncorrelated with the conventional index of tumour proliferation and with the density of replicating cells in the tissue.

We then verified if the value distribution of each feature was different between AC and LCNEC tumours. The Mann–Whitney U test deemed 93 features as coming from the same statistical distribution across the two histologic subtypes, i.e., whose value in a tumour does not seem to depend on the histologic type. Out of 93, 57 also comprised the 87 uncorrelated with both Ki-67 LI and Ki-67^+^ cell density. Moreover, 5 of these 57 belong to the 18 most prognostically relevant, in an almost perfect overlap with the six also uncorrelated with the two Ki-67 indices. Notably, they include the three features residing in the top six, meaning that the 50% of top prognostic features that are uncorrelated with the proliferation indices are exactly those that are also histology independent.

**Figure 5:**
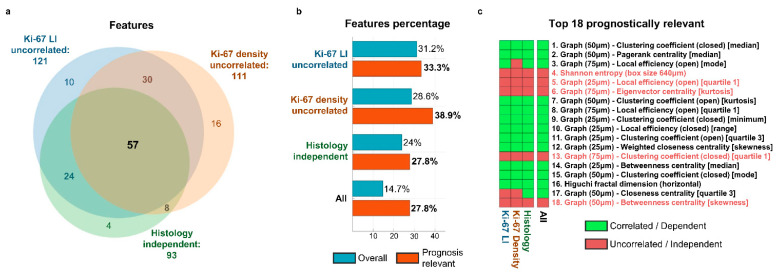
(**a**) Venn diagram depicting the relationships and overlaps between three relevant sets of features: those uncorrelated with Ki-67 LI (121, in total), the ones uncorrelated with the density of Ki-67^+^ cells (111) and the histology-independent ones (93). (**b**) Percentage of features belonging to each of the three mentioned feature sets and to their intersection. Blue bars indicate the percentage among the total 388 features remaining after preliminary filtering, red bars the percentage in the 18 most prognostically relevant. (**c**) List of the 18 most prognostically relevant features. The corresponding heatmap shows whether they belong to any of the previously presented feature sets (in red) or not (in green).

Figure 5 presents a recap of these results. In summary, 57 features out of 388 (14.7%) appear to be both histology independent and uncorrelated with Ki-67 LI and Ki-67^+^ cell density. Most importantly, this category of features is highly over-represented in the prognosis-relevant ones: 5 out of 18 (27.8%), with three among the six most relevant overall.

## 3. Discussion

We proposed a tumour grading framework based on a quantitative description of the spatial pattern displayed by cells positive to the Ki-67 proliferative marker. We developed an automated pipeline that analyses the entire extent of whole slide images of resection tissue and computes objective descriptors, requiring minimal manual pre-processing. We successfully predicted the prognostic outcome in the most controversial lung NENs in a histology-independent way, outperforming the classification based on the conventional Ki-67 Labelling Index and on the density of Ki-67^+^ cells in the tissue. Our solution overcomes the well-known limitations of gold standard grading techniques for lung NENs and represents a proposal for a whole new approach to their grading, potentially extendable to other NENs and in line with the recent invitation of World Health Organization experts to question the traditional concept of grading for these neoplasms [16].

**Figure 6:**
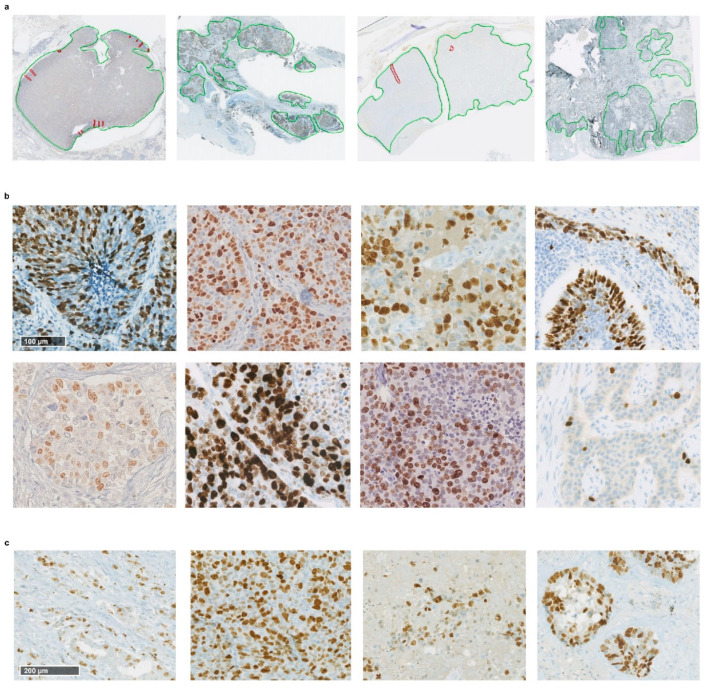
(**a**) Examples of outlined WSIs from our dataset. Each presents a different number of outlined regions, with unique shapes and sizes. (**b**) Examples of hue and texture variability between samples. Each image presents a unique landscape, with peculiar colouring and cell disposition patterns. (**c**) Variability of texture and cellular organisation patterns within the same WSI.

Despite the multi-centre dataset, our system proved capable of successfully dealing with WSIs’ heterogeneity on different levels, a challenge strongly enhanced by this multiplicity of sources. This capability constitutes a fundamental feature in a domain where heterogeneity of samples is a key factor that limits repeatability of grade assessment. Figure 6 provides a series of examples of these different layers of heterogeneity, present both between and within WSIs. First, each WSI is unique in terms of shape and number of neoplastic regions in the tissue section (Figure 6a). Because our system derives features that quantify characteristics independent of regions’ shape and size, it is not affected by this variability. Instead, it even exploits heterogeneity at this level, since morphological and textural differences between regions might encode relevant prognostic information. The second and perhaps most challenging heterogeneity factor lies in the variability of colouring and texture between WSIs. Each tissue section is, per se, a unique specimen in terms of morphology, with its own array of cell shapes, sizes and distribution patterns, and antibody staining introduces the additional variable of hue. As the staining procedure is performed manually, variability in colouring can already be observed between samples processed in the same laboratory, especially concerning staining intensity. Furthermore, different laboratories likely employ different immunohistochemical protocols to perform the procedure—i.e., different primary antibody clones, staining platforms, etc. All these elements are recognised to lead to radically diverse results in terms of visual appearance of the WSIs [33,34], as clearly exemplified in Figure 6b. The capability of dealing with such heterogeneous samples demonstrated by our framework represents one of its core strengths. This flexibility is essential when working with rare tumours, since the need to employ multi-centre data is fundamentally unavoidable. Lastly, Figure 6c provides an example of texture and distribution pattern variability within a single tissue slide. This variability, in addition to representing another challenge in the correct recognition of stained cells throughout the entirety of WSIs, provides even further evidence on how limiting it is to exclusively consider a single 0.4 mm^2^ region—the current consensus proposal [16]—out of all the available tissue for grading, as an unreckonable amount of potentially useful information is completely neglected.

In summary, our framework not only overcomes the most relevant challenge posed by Ki-67, but turns it into the foundation of the tumour’s characterisation task, and the results achieved in light of the many potential issues highlight the validity and soundness of this approach.

**Figure 7:**
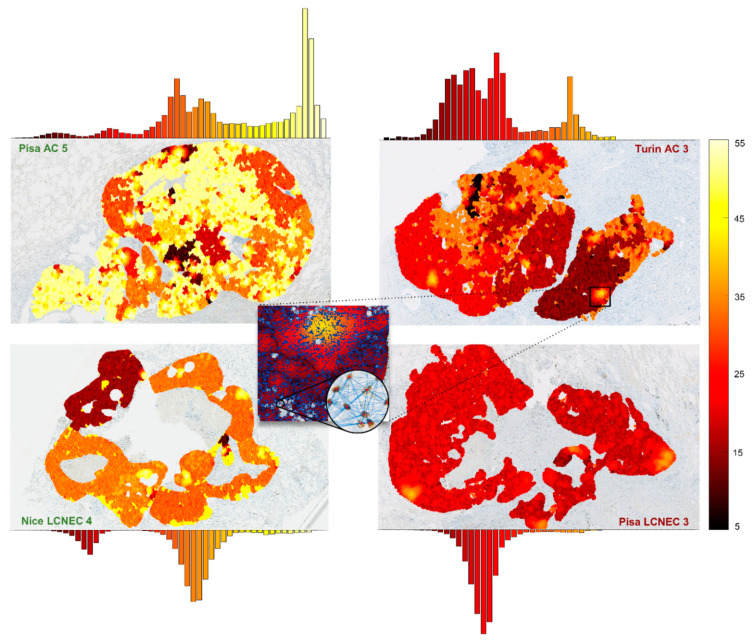
Eigenvector centrality value of each node in four samples in respective 75 µm graphs. The samples presented include two GP, name in green, and two PP, name in red. Each image is paired with the corresponding histogram of centrality values. Higher values—i.e., lighter colour shades—indicate a greater relevance of the node in the network’s topology. For visualisation purposes, the measure presented is a transformation of the original eigenvector centrality value *c_eig_*, obtained for each node as *c’_eig_* = *log_2_* (*c_eig_*) *– min* (*log_2_*(*c_eig_*)), with the minimum function computed over all nodes of that graph.

For our model, we employed a ranking-based feature selection method, rather than one yielding a fixed and unordered set of supposedly relevant parameters, to maximise the possibility of gaining meaningful insight from the results. As previously shown, among the most prognosis-relevant ones, we obtained features from the information theory and fractality groups, despite these categories including just four and six features in total, respectively, out of 490. Specifically, Higuchi’s horizontal fractal dimension and Shannon’s entropy for windows of 640 µm^2^ were significant. The former suggests the presence of fractality patterns in the distribution of the cells, while the latter—which ranked fourth overall—deems relevant the variability of relative concentration of Ki-67^+^ cells in equal-sized areas of tissue, clearly pointing out a different perspective with respect to accounting for the concentration in a single region. The remaining significant features belong to the graph theory category, with parameters referring to all the three different connection radiuses evaluated, a result that confirms how looking at the cell network’s organisation at different scales did provide relevant insight. These features include a variety of summary statistical indices computed over three sub-categories of graph parameters: centralities, clustering coefficients and efficiencies, all nontrivial indicators of the disposition of the nodes. This heterogeneity within the relevant graph theory features, together with the presence in the ranking of the two features from the small-sized fractality and information theory categories, represents a success for the proposed multi-perspective investigation approach of the replicating cell topology. Combining different techniques to tackle the grading task appears to be viable and effective. We wish, however, to underline how intricate the problem remains, and how nontrivial the interpretation of the results can be. As an example, Figure 7 shows the distribution of eigenvector centrality values for 75 µm graphs in four samples, two GP (one AC, one LCNEC) and two PP (same). The kurtosis of the presented distribution emerged as the sixth most relevant feature in discerning prognostic class among the total 490. This feature can be viewed as a measure of how evenly Ki-67^+^ cells are distributed throughout the tissue. Higher values indicate a rather uniformly spaced distribution of the cells, while lower ones denote some variability, i.e., the presence of regions with different degrees of intra and/or interconnection. As exemplified in Figure 7, this appears to be often due to large, well-circumscribed areas where nodes present visibly different centrality scores, rather than a general, more scattered unevenness. However, although patterns like this can be observed, for other features as well, we could not identify for any individual feature a defining trait that might appear to provide separation between GP and PP samples by means of that feature alone. Instead, the message that clearly transpires from our observations is that characterising the neoplasm exhaustively enough to assess its prognosis is a rather complex problem, whose solution appears far beyond the capability of either the most comprehensive or specific individual parameter, and therefore in turn of any limited marker such as the traditional Ki-67 LI.

In addition to these considerations, our statistical analyses did raise an interesting point that we wish to highlight. We showed how a consistent fraction of the features were not correlated with both Ki-67 LI and Ki-67^+^ cell density—and, therefore, with the number of replicating cells in the neoplasm, either in relation to the total number of cells or to the tissue area—and how these features are largely the same as those that are histology independent. This is particularly clear in the top 18 prognosis-relevant features shown in the heatmap of Figure 5c, where just seven features belong to at least one of the subsets, but five belong to all three. It strikes us as remarkably interesting not only this large overlap, but especially the strong over-representation of this category of features among those that resulted most relevant in discerning between good and poor prognosis, up to the outstanding 50% in the top six. These findings not only provide evidence towards the existence of organisation patterns that appear to exhibit regardless of the current histological categorisation, and independently of how intense the proliferation activity is, but most of all suggest that the analysis and quantification of these patterns, in particular, might be rather relevant in assessing the prognosis of lung NENs. Moreover, this seeming independence from histology of such prognosis-relevant patterns encourages investigation of their presence even in NENs of other sites of the body, leaving the door open to the potential pursuit of a unified grading system.

## 4. Materials and Methods

### 4.1. Data Acquisition

Formalin-fixed and paraffin-embedded tumour tissue was used in all cases. Ki-67 antigen immunoreactivity and the Ki-67 Labelling Index (expressed as percentage of immunoreactive cells on hot spot areas of ≥2000 tumour cells or 2 mm^2^) were devised as previously detailed [35]. A single, representative section was employed for each tumour case, checked by a pathologist regarding the presence, amount and viability of tumour cells. WSIs were obtained by scanning tumour sections through Hamamatsu Nano-Zoomer XR (Japan) at 40× magnification, with the aid of an automated focus system.

The study was authorised by the internal ethical committee of MultiMedica (Milan, Italy), with the registration number CE-40.2018/Prot 369.2019 and under the title “Dissecting the biological meaning of intratumour heterogeneity of the Ki-67 proliferation antigen in lung neuroendocrine tumours by means of machine learning tools on histological images from virtual slides—IMMINENT (Image Mining for Lung Neuroendocrine Tumours)”. Informed consent was obtained from all human research participants at admission.

### 4.2. Tumour Regions Outlining

The outlining of tumour regions on WSIs was performed manually using Hamamatsu’s proprietary software NDP.View2 [32]. This process simply consists of the rough delimitation by means of hand-drawn lines of the regions of the slide containing viable neoplastic tissue, in green, and possible areas that should be excluded from the analysis—due to the presence of necrosis, cutting artefacts, inflammatory infiltrate, granulation tissue or fibrosis—in red. WSIs with overlaid demarcation lines were then exported as images in bitmap format with either 20× or 10× magnification level (corresponding to 0.454 µm/pixel and 0.908 µm/pixel resolutions, respectively), the highest allowed by NDP.View2 depending on the size of the image being exported.

### 4.3. Image Rotation and Framing

For each image, a mask is created according to the previously outlined areas: regions not surrounded by a green line (i.e., background) and regions surrounded by a red line (i.e., to be neglected) are set to black. The image is then rotated in order to make the major axis of the ellipse that has the same second moment as the non-black regions of the image lie horizontally. The angle of this axis is obtained through the MATLAB function regionprops, with argument “Orientation”. This operation is carried out to make sure that the starting point of the processing pipeline is the same independently on the slide rotation and export frame on the viewer from which the bitmap image is obtained, ensuring repeatability of the whole process. The frame of the rotated image is then shrunk to its minimum possible size, i.e., set to the smallest rectangle containing all the non-black regions, by cutting out black borders.

### 4.4. Ki-67^+^ Nuclei Identification

First, 2D median and Gaussian filtering are sequentially applied to the image to perform noise reduction and image smoothing. The kernels employed measure 5 × 5 pixels for 20× images and 3 × 3 pixels for 10× images, with a standard deviation of 0.5 for the Gaussian one. The first stage of the identification process is then carried out at the individual pixel level, detecting brown shaded ones by means of thresholding on RGB channels of the image. The upper threshold on the blue channel is the only parameter that differs between images, to account for hue variability, while every other threshold value is fixed. A binary black and white version of the original image is produced as output of this stage. White pixels are those selected by the thresholding process, i.e., potentially belonging to Ki-67-positive cell nuclei, while every other pixel is set to black. Box blurring—5 × 5 kernel for 20× images, 3 × 3 for 10×—and filtering of isolated white pixels are applied to this binary image for further smoothing and noise reduction. This step is followed by a filling operation, setting to white any black area fully surrounded by white pixels. Watershed segmentation is then performed to identify and split candidate adjacent nuclei that ended up belonging to the same white continuum. Finally, after a last size-based filtering operation, discarding any aggregate considered likely too small or too big to be a cell nucleus, an (*x,y*) coordinate pair describing its location within the image is assigned to the centroid of each identified positive nucleus.

### 4.5. Features Computation

Four categories of features are computed for each image, basing on the locations of the identified Ki-67-positive cells, for a total of 490 parameters. The full list is available in Table S2, and the feature computation procedures that are not described in detail in this section are available in Methods S1.

### 4.6. Graph Theory Features

A graph is constructed by considering positive cells as nodes and connecting those within a chosen proximity threshold from one another through edges. Three different thresholds are employed, leading to three different graphs: 25, 50, and 75 µm. From each graph, two sets of features describing its topology are derived: global and local. Global features are those summarising a certain property for the entire graph in a single numerical value, while local features are single node descriptors, i.e., indices computed for each node of the graph individually, quantifying some characteristic of the node in relation to the rest of the graph. To obtain a summarisation of the behaviour of local features throughout the whole graph, a set of 16 statistical indices are computed over each of them, and these aggregate values are retained as the actual features for the image, in addition to the global ones. The employed statistical indices are arithmetic, geometric and harmonic mean, standard deviation, skewness, kurtosis, range, mode, minimum, maximum, first, second and third quartiles, and second, third and fourth central moments.

### 4.7. Stochastic Geometry Features

The distribution of positive nuclei is interpreted as a spatial point process. Process intensity, Ripley’s K function estimation for a stationary process and the corresponding L function [36] are computed for an array of radiuses between 5 and 50 µm, with a step of 2.5 µm.

### 4.8. Information Theory Features

The image is partitioned into adjacent squared windows of equal size. For each bin falling completely inside nonblack regions of the slide, the number *n* of positive cells in it is counted, and each of these numbers represents an occurrence of the event *e* defined as “there is a window containing *n* Ki-67^+^ cells in the image”. Shannon entropy is then computed as *H* =∑*_e_*(−log p_e_* *p_e_*), where *p_e_* is the empirical probability of event *e*, obtained as the number of occurrences of *e* out of all the events registered. This feature is computed for windows of side 20, 40, 80, 160, 320, and 640 µm.

### 4.9. Fractality Features

The Hausdorff fractal dimension of the positive cell distribution is computed according to the box-counting algorithm [37]. Minimum box size is set to 10 µm, maximum to 1100, and a straight line is used as the fitting curve. Horizontal and vertical fractal dimensions of the distribution of Ki-67^+^ cells are computed according to a 2D generalisation of Higuchi’s method [38], and their average is included as an additional feature.

### 4.10. Feature Selection

First, all the features having the same value in all the images of the considered dataset are discarded. A correlation-based filtering step is then performed to filter out plainly redundant features before performing the major feature selection. Pearson’s correlation coefficient is computed between every possible pair of features, and for each pair with a correlation equal to one in absolute value, the feature with the highest variance within the dataset is kept while the other is discarded.

The last feature selection step employs Neighbourhood Component Analysis [39], in its MATLAB implementation from the Statistics and Machine Learning Toolbox, namely, the fsnca function. NCA outputs a weight value for each feature of the input dataset according to its estimated relevance in discriminating good and poor prognosis samples, evaluated through a multivariate analysis. The value of the algorithm’s regularisation parameter lambda is selected among a range of possible values determined according to the size of the dataset, by comparing the average losses obtained in 100 repetitions of 5-fold cross validation. Each model is fitted using stochastic gradient descent with a limit of 30 iterations and data standardisation (z-score) enabled. Once the optimal lambda is found, NCA is performed over the whole dataset with Limited memory Broyden–Fletcher–Goldfarb–Shanno (LBFGS) [40] solver and data standardisation (z-score) enabled, providing in output a weight value for each feature. Features are then filtered according to a cut-off weight, which is set to 0.01; features weighing less than the cut-off are discarded.

### 4.11. Predictors

Two classification algorithms are compared: k-Nearest Neighbours (k-NN), for k = 3, and Support Vector Machines (SVM). k-NN models are fitted employing Euclidean distance metric, squared inverse distance weighting, “exhaustive” nearest neighbour search method, “nearest” as tie-breaking policy and data standardisation (z-score) enabled. SVM models are fitted employing a polynomial kernel, Iterative Single Data Algorithm (ISDA) optimisation routine [41] and data standardisation (z-score) enabled. Model performance metrics are computed as the average over 100 repetitions of 5-fold cross-validation.

### 4.12. Feature Analysis

Correlation analyses between our features, Ki-67 LI and Ki-67^+^ cell density, were carried out employing the Pearson correlation coefficient. Difference in distribution of feature values between AC and LCNEC samples was evaluated through a Mann–Whitney U test. Multiple testing correction was applied to each batch of tests according to the Benjamini–Hochberg procedure, yielding the corresponding False Discovery Rate for each p-value. Results were then considered statistically significant if FDR < 0.05.

### 4.13. Implementation Details

All the software was implemented in MATLAB R2018b with the exception of the scripts for deriving stochastic geometry features, which were implemented in R 3.3.0. Computations were performed on a Dell Precision 5820 Tower equipped with an Intel Xeon W-2123 CPU at 3.6 GHz and 64 GB of RAM, running Windows 10 as the operating system.

## 5. Conclusions

In this work, we proposed a new approach to the interpretation of the well-recognised Ki-67 marker for grading lung neuroendocrine neoplasms. We proposed an automated framework that analyses the spatial organisation of Ki-67-positive cells on the extent of whole-slide tissue images, exploiting the otherwise inconvenient heterogeneous distribution of this marker as a valuable source of information. We show that the subsequent machine learning-based prediction of prognosis outperforms the results obtained by the well-recognised Ki-67 Labelling Index and the density of Ki-67^+^ cells in the tissue.

The proposed approach to grading overcomes the limitations of more traditional techniques, disclosing a possible path for the future of this procedure. Moreover, it allowed us to detect patterns that suggest not only the existence but also the high prognostic significance of organisation traits that seem to exhibit regardless of tumour type and of the apparent intensity of the proliferation activity. As such, these patterns could potentially be shared by other NENs, encouraging the investigation of their presence outside of the lung, and providing hope for the potential pursuit of a unified grading system.

## Data Availability

The data that support the findings of this study are available on reasonable request from the corresponding author.

## Supplementary Materials

The following are available online at https://www.mdpi.com/article/10.3390/cancers13194875/s1, Table S1: Computational Data, Table S2: Complete List of Features, Methods S1: Supplementary Methods.

## References

[1] Surveillance, Epidemiology, and End Results (SEER) Program (Www.Seer.Cancer.Gov) SEER*Stat Database: Incidence—SEER Research Limited-Field Data, 21 Registries, November 2019 Sub (2000–2017)—Linked To County Attributes—Time Dependent (1990–2017) Income/Rurality, 1969–2018 Counties, National Cancer Institute, DCCPS, Surveillance Research Program, Released April 2020, Based on the November 2019 Submission. https://seer.cancer.gov/.

[2] Sackstein P.E., O’Neil D.S., Neugut A.I., Chabot J., Fojo T. (2018). Epidemiologic trends in neuroendocrine tumors: An examination of incidence rates and survival of specific patient subgroups over the past 20 years. Semin. Oncol..

[3] Travis W.D., Brambilla E., Nicholson A.G., Yatabe Y., Austin J.H.M., Beasley M.B., Chirieac L.R., Dacic S., Duhig E., Flieder D.B. (2015). The 2015 World Health Organization Classification of Lung Tumors: Impact of Genetic, Clinical and Radiologic Advances since the 2004 Classification. J. Thorac. Oncol..

[4] Pelosi G., Pattini L., Morana G., Fabbri A., Faccinetto A., Fazio N., Valeri B., Sonzogni A. (2017). Grading lung neuroendocrine tumors: Controversies in search of a solution. Histol. Histopathol..

[5] Alcala N., Leblay N., Gabriel A., Mangiante L., Hervas D., Giffon T., Sertier A.S., Ferrari A., Derks J., Ghantous A. (2019). Integrative and comparative genomic analyses identify clinically relevant pulmonary carcinoid groups and unveil the supra-carcinoids. Nat. Commun..

[6] George J., Walter V., Peifer M., Alexandrov L.B., Seidel D., Leenders F., Maas L., Müller C., Dahmen I., Delhomme T.M. (2018). Integrative genomic profiling of large-cell neuroendocrine carcinomas reveals distinct subtypes of high-grade neuroendocrine lung tumors. Nat. Commun..

[7] Cros J., Théou-Anton N., Gounant V., Nicolle R., Reyes C., Humez S., Hescot S., De Montpréville V.T., Guyétant S., Scoazec J.-Y. (2020). Specific Genomic Alterations in High-Grade Pulmonary Neuroendocrine Tumours with Carcinoid Morphology. Neuroendocrinology.

[8] Simbolo M., Barbi S., Fassan M., Mafficini A., Ali G., Vicentini C., Sperandio N., Corbo V., Rusev B., Mastracci L. (2019). Gene Expression Profiling of Lung Atypical Carcinoids and Large Cell Neuroendocrine Carcinomas Identifies Three Transcriptomic Subtypes with Specific Genomic Alterations. J. Thorac. Oncol..

[9] Kasajima A., Konukiewitz B., Oka N., Suzuki H., Sakurada A., Okada Y., Kameya T., Ishikawa Y., Sasano H., Weichert W. (2019). Clinicopathological Profiling of Lung Carcinoids with a Ki67 Index > 20. Neuroendocrinology.

[10] Marchiò C., Gatti G., Massa F., Bertero L., Filosso P., Pelosi G., Cassoni P., Volante M., Papotti M. (2017). Distinctive pathological and clinical features of lung carcinoids with high proliferation index. Virchows Arch..

[11] Quinn A.M., Chaturvedi A., Nonaka D. (2017). High-Grade Neuroendocrine Carcinoma of the Lung with Carcinoid Morphology: A Study of 12 Cases. Am. J. Surg. Pathol..

[12] Simbolo M., Mafficini A., O’Sikora K., Fassan M., Barbi S., Corbo V., Mastracci L., Rusev B., Grillo F., Vicentini C. (2017). Lung neuroendocrine tumours: Deep sequencing of the four World Health Organization histotypes reveals chromatin-remodelling genes as major players and a prognostic role for TERT, RB1, MEN1 and KMT2D. J. Pathol..

[13] Rekhtman N., Pietanza M.C., Hellmann M.D., Naidoo J., Arora A., Won H., Halpenny D.F., Wang H., Tian S.K., Litvak A.M. (2016). Next-Generation Sequencing of Pulmonary Large Cell Neuroendocrine Carcinoma Reveals Small Cell Carcinoma–like and Non–Small Cell Carcinoma–like Subsets. Clin. Cancer Res..

[14] Oka N., Kasajima A., Konukiewitz B., Sakurada A., Okada Y., Kameya T., Weichert W., Ishikawa Y., Suzuki H., Sasano H. (2020). Classification and Prognostic Stratification of Bronchopulmonary Neuroendocrine Neoplasms. Neuroendocrinology.

[15] Rindi G., Klersy C., Inzani F., Fellegara G., Ampollini L., Ardizzoni A., Campanini N., Carbognani P., De Pas T.M., Galetta D. (2014). Grading the neuroendocrine tumors of the lung: An evidence-based proposal. Endocr. Relat. Cancer.

[16] Rindi G., Klimstra D.S., Abedi-Ardekani B., Asa S.L., Bosman F., Brambilla E., Busam K.J., De Krijger R.R., Dietel M., El-Naggar A.K. (2018). A common classification framework for neuroendocrine neoplasms: An International Agency for Research on Cancer (IARC) and World Health Organization (WHO) expert consensus proposal. Mod. Pathol..

[17] Brcic L., Heidinger M., Sever A.Z., Zacharias M., Jakopovic M., Fediuk M., Maier A., Quehenberger F., Seiwerth S., Popper H. (2019). Prognostic value of cyclin A2 and B1 expression in lung carcinoids. Pathology.

[18] Del Gobbo A., Vaira V., Rocco E.G., Palleschi A., Bulfamante G., Ricca D., Fiori S., Bosari S., Ferrero S. (2014). The Oncofetal Protein IMP3: A Useful Marker to Predict Poor Clinical Outcome in Neuroendocrine Tumors of the Lung. J. Thorac. Oncol..

[19] Neubauer E., Wirtz R.M., Kaemmerer D., Athelogou M., Schmidt L., Sänger J., Lupp A. (2016). Comparative evaluation of three proliferation markers, Ki-67, TOP2A, and RacGAP1, in bronchopulmonary neuroendocrine neoplasms: Issues and prospects. Oncotarget.

[20] Altinay S., Metovic J., Massa F., Gatti G., Cassoni P., Scagliotti G.V., Volante M., Papotti M. (2019). Spread through air spaces (STAS) is a predictor of poor outcome in atypical carcinoids of the lung. Virchows Arch..

[21] Aly R.G., Rekhtman N., Li X., Takahashi Y., Eguchi T., Tan K.S., Rudin C.M., Adusumilli P.S., Travis W.D. (2019). Spread Through Air Spaces (STAS) Is Prognostic in Atypical Carcinoid, Large Cell Neuroendocrine Carcinoma, and Small Cell Carcinoma of the Lung. J. Thorac. Oncol..

[22] Asa S., Volynskaya Z., Mete O., Pakbaz S., Al-Ghamdi D. (2019). Ki67 quantitative interpretation: Insights using image analysis. J. Pathol. Inform..

[23] Pelosi G., Sonzogni A., Harari S., Albini A., Bresaola E., Marchiò C., Massa F., Righi L., Gatti G., Papanikolaou N. (2017). Classification of pulmonary neuroendocrine tumors: New insights. Transl. Lung Cancer Res..

[24] Warth A., Fink L., Fisseler-Eckhoff A., Jonigk D., Keller M., Ott G., Rieker R.J., Sinn P., Söder S., Soltermann A. (2013). Interobserver Agreement of Proliferation Index (Ki-67) Outperforms Mitotic Count in Pulmonary Carcinoids. Virchows Arch..

[25] Kwon A.-Y., Park H.Y., Hyeon J., Nam S.J., Kim S.W., Lee J.E., Yu J.-H., Lee S.K., Cho S.Y., Cho E.Y. (2019). Practical approaches to automated digital image analysis of Ki-67 labeling index in 997 breast carcinomas and causes of discordance with visual assessment. PLoS ONE.

[26] Yang Z., Tang L.H., Klimstra D.S. (2011). Effect of Tumor Heterogeneity on the Assessment of Ki67 Labeling Index in Well-differentiated Neuroendocrine Tumors Metastatic to the Liver: Implications for Prognostic Stratification. Am. J. Surg. Pathol..

[27] Boland J.M., Kroneman T.N., Jenkins S.M., Terra S.B., Xie H., Molina J., Mounajjed T., Roden A.C. (2020). Ki-67 Labeling Index in Pulmonary Carcinoid Tumors: Comparison Between Small Biopsy and Resection Using Tumor Tracing and Hot Spot Methods. Arch. Pathol. Lab. Med..

[28] Pham D.-T., Skaland I., Winther T.L., Salvesen Ø., Torp S.H. (2020). Correlation Between Digital and Manual Determinations of Ki-67/MIB-1 Proliferative Indices in Human Meningiomas. Int. J. Surg. Pathol..

[29] Geread R., Morreale P., Dony R.D., Brouwer E., Wood G.A., Androutsos D., Khademi A. (2019). IHC Color Histograms for Unsupervised Ki67 Proliferation Index Calculation. Front. Bioeng. Biotechnol..

[30] Dessauvagie B., Thomas A., Thomas C., Robinson C., Combrink M., Budhavaram V., Kunjuraman B., Meehan K., Sterrett G., Harvey J. (2019). Invasive lobular carcinoma of the breast: Assessment of proliferative activity using automated Ki-67 immunostaining. Pathology.

[31] Hida A.I., Omanovic D., Pedersen L., Oshiro Y., Ogura T., Nomura T., Kurebayashi J., Kanomata N., Moriya T. (2020). Automated assessment of Ki-67 in breast cancer: The utility of digital image analysis using virtual triple staining and whole slide imaging. Histopathology.

[32] NDP.View2 Viewing Software. https://nanozoomer.hamamatsu.com/eu/en/product/search/U12388-01/index.html.

[33] Blank A., Wehweck L., Marinoni I., Boos L., Bergmann F., Schmitt A.M., Perren A. (2015). Interlaboratory variability of MIB1 staining in well-differentiated pancreatic neuroendocrine tumors. Virchows Arch..

[34] Røge R., Nielsen S., Riber-Hansen R., Vyberg M. (2019). Impact of Primary Antibody Clone, Format, and Stainer Platform on Ki67 Proliferation Indices in Breast Carcinomas. Appl. Immunohistochem. Mol. Morphol..

[35] Fabbri A., Cossa M., Sonzogni A.M., Papotti M., Righi L., Gatti G., Maisonneuve P., Valeri B., Pastorino U., Pelosi G. (2017). Ki-67 labeling index of neuroendocrine tumors of the lung has a high level of correspondence between biopsy samples and surgical specimens when strict counting guidelines are applied. Virchows Arch..

[36] Dixon P. (2001). Ripley’s K Function. Encycl. Env..

[37] Napolitano A., Ungania S., Cannat V. (2012). Fractal Dimension Estimation Methods for Biomedical Images. MATLAB—A Fundamental Tool for Scientific Computing and Engineering Applications—Volume 3.

[38] Ahammer H. (2011). Higuchi Dimension of Digital Images. PLoS ONE.

[39] Yang W., Wang K., Zuo W. (2012). Neighborhood Component Feature Selection for High-Dimensional Data. J. Comput..

[40] Nocedal J. (1980). Updating Quasi-Newton Matrices with Limited Storage. Math. Comput..

[41] Kecman V., Huang T.-M., Vogt M. (2005). Iterative Single Data Algorithm for Training Kernel Machines from Huge Data Sets: Theory and Performance. Integr. Fuzzy Logic. Chaos Theory.

