# Supplementary material for: Automated Analysis of Proliferating Cells Spatial Organisation Predicts Prognosis in Lung Neuroendocrine Neoplasms
Source: Cancers (Basel). 2021 Sep 29;13(19):4875. doi: 10.3390/cancers13194875 (PMC8508355; doi:10.3390/cancers13194875)
Supplement: Supplementary file 1 [file cancers-13-04875-s001.zip › Supplementary Methods S1.pdf]

Supplementary methods

Features computation

Graph theory

Terminology

In general, when formally defining parameters, G is used to indicate the graph, v the nodes and e the edges. Additionally:

- **sub-graph of a node v_i:** set of nodes directly connected to v_i. A closed sub-graph includes v_i itself, an open one does not.
- **connected component (or node set):** set of nodes in which any two nodes are connected to each other by paths, and that is not connected to any additional vertices in the rest of the graph.

General features

Local parameters

Name	Definition
Degree	Number of edges of a node v_i.

Global parameters

Name	Definition
Number of edges	Total number e of edges in the graph.
Number of nodes	Total number n of nodes in the graph.
% of unconnected nodes	Percentage of nodes with degree = 0.
% of end nodes	Percentage of nodes with degree = 1.
Size of largest connected component normalized	Number of nodes in the largest connected component normalized by the total number of nodes of the graph.
Number of connected component normalized	Normalized by the total number of nodes of the graph.
Average number of nodes in a connected component normalized	Normalized by the total number of nodes of the graph.

Path length features

The path length $d(v_i, v_j)$ between two nodes v_i and v_j is defined as the minimum number of edges to be traversed to go from v_i to v_j. By definition, $d_{v_i,v_j} = inf$ if v_i and v_j are not connected. However, for simplification purposes, it has been considered for these cases $d_{v_i,v_j} = 0$ for the first two definitions of the table.
The weighted path length $\hat{d}(v_i, v_j)$ is instead defined as the minimum distance separating v_i and v_j, i.e., the path length computed on a graph with weighted edges. In a weighted graph the path length between any two adjacent nodes is not equal to the fixed value of 1, but is instead obtained through a certain distance measure. In our case, the measure employed is the Euclidean distance between the two Ki-67+ nuclei corresponding to v_i and v_j on the whole-slide image of the tumoral tissue.

Global parameters

Index name	Formal definition	Description
Average shortest path length between 2 nodes	$d_{av} = \frac{2}{n(n-1)} \sum_{i \neq j} d(v_i, v_j)$	$\frac{n(n-1)}{2}$ is the number of node pairs in a graph.
Maximum shortest path length between 2 nodes	$d_{max} = max(d(v_i, v_j))$, $i \neq j$.	
% of unconnected node pairs	$NCP = \frac{2}{n(n-1)} \#d_{inf}$	d_{inf} is any node pair of G such that $d_{i,j} = inf$.

Centralities

Centrality values assign to each node a score of relative importance within the graph v_i.

Local parameters

Index name	Formal definition	Description
Closeness centrality	$C_C(v_i) = \frac{1}{\#ns_{v_i} - 1} \sum_{v_j \in ns_{v_i} \setminus v_i} d(v_i, v_j)$, where ns_{v_i} is the node set containing v_i and $\#$ represents its cardinality.	Inverse of the sum of path lengths $d(v_i, v_j)$ between v_i and each other node v_j belonging to the same connected component, normalized by the size of such connected component - excluding v_i.
Weighted closeness centrality	$C_W(v_i) = \frac{1}{\#ns_{v_i} - 1} \sum_{v_j \in ns_{v_i} \setminus v_i} \hat{d}(v_i, v_j)$, where $\hat{d}(v_i, v_j)$ represents the weighted path length between v_i and v_j.	As closeness centrality, but computed on the weighted graph, i.e., considering weighted path lengths.
Betweenness centrality	$C_B(v_i) = \sum_{j,k \neq i} \frac{n_{v_j,v_k}(v_i)}{N_{v_j,v_k}}$, where $n_{v_j,v_k}(v_i)$ is the number of shortest paths from j to k traversing v_i and N_{v_j,v_k} is the total number of shortest paths from v_j to v_k.	Measures how often the node v_i appears on the shortest paths between any two nodes of the graph. This centrality was computed on the weighted graph.

Pagerank centrality	$C_{PR}(v_i) = \frac{1-d}{n} + d\left(\sum_{v_j \in SG_{v_i}} \frac{C_{PR(v_j)}}{D_{v_j}}\right)$, where SG_{v_i} is the open sub-graph of v_i, $C_{PR}(v_j)$ and D_{v_j} are respectively the pagerank centrality and the grade of v_j, and d is the follow probability, or damping factor, set to 0.85.	This score quantifies the average time spent on a node v_i during a random walk throughout the graph.
Eigenvector centrality	$C_{EV}(v_i) = \frac{1}{\lambda_n} \sum_{v_j \in G} A_{i,j}\, C_{EV}(j)$, where λ_n is the maximum eigenvalue of the adjacency matrix A.	This measure quantifies the relevance of v_i considering that a connection to a high-scoring node is more relevant than a connection to a low-scoring one.

Efficiencies

Efficiency is defined as the inverse of path length: $Eff_{v_i,v_j} = \frac{1}{d(v_i, v_j)}$. Efficiencies have been computed on unweighted graphs. Local efficiency features have been computed for both open and closed sub-graphs.

Local parameters

**Index name**	**Formal definition**	**Description**
Local efficiency	$Eff_{v_i,v_j} = \sum_{v_j \in SG_{v_i}} \frac{1}{d(v_i, v_j)}$, where SG_{v_i} is the - open or closed - sub-graph of v_i.	Efficiency computed individually on the subgraph of a node.

Global parameters

**Index name**	**Formal definition**	**Description**
Global efficiency	$Eff = \frac{2}{n\,(n-1)} \sum_{i \neq j} \frac{1}{d\,(v_i, v_j)}$.	Average efficiency between each pair of nodes of the graph.

Clustering coefficient

Computed considering either open or closed sub-graphs.

Local parameters

**Index name**	**Formal definition**	**Description**
Clustering coefficient	$CC(v_i) = \frac{2}{n_{SG_{v_i}}(n_{SG_{v_i}}-1)} e_{SG_{v_i}}$, where $n_{SG_{v_i}}$ is the number of nodes in the sub-graph of v_i and $e_{SG_{v_i}}$ the number of edges.	Actual number of edges in the sub-graph divided by the maximum possible number of edges that could be present - i.e., by the number of node pairs in the sub-graph.

Spectral features

Introducing the following additional definitions:

- **adjacency matrix A:** matrix of [number of nodes in G x number of nodes in G] used to represent G. $A_{i,j}$ is equal to 1 if an edge directly connects v_i and v_j, 0 otherwise.
- **Laplacian matrix L:** matrix of [number of nodes in G x number of nodes in G] used to represent G. $L_{i,i}$ is equal to the degree of v_i, $L_{i,j}, i \neq j$ equals -1 if an edge directly connects v_i and v_j, 0 otherwise.
- **normalized Laplacian matrix L_{norm}:** matrix of [number of nodes in G x number of nodes in G] used to represent G. $L_{norm_{i,i}}$ is equal to 1 if the degree of v_i is greater than zero, 0 otherwise; $Lnorm_{i,j}, i \neq j$ equals $\frac{-1}{\sqrt{degree_{v_i} * degree_{v_j}}}$ if an edge directly connects v_i and v_j, 0 otherwise.

Global parameters

**Index name**	**Formal definition**	**Description**
Largest eigenvalue (A)	λ_n	Largest eigenvalue of the adjacency matrix A.

Second largest eigenvalue (A)	λ_{n-1}	
Spectral gap	$\Delta\lambda = \lambda_n - \lambda_{n-1}$	Difference between the largest and the second largest eigenvalues.
Graph energy	$E = \sum_{i=1}^{n} \lambda_i$	Also called energy of A, it is the sum of all A eigenvalues.
Laplacian energy	$LE = \sum_{i=1}^{n} \left\| \mu_i - \frac{2e}{n} \right\|$, where μ_i is the $i-th$ eigenvalue of the Laplacian matrix L.	Laplacian energy depends on L's eigenvalues and on the total number of edges and nodes of the graph.
Triangles number	$N_{triangles} = \sum_{i=1}^{n} \lambda_i^3$	
Number of ones	$\#(\mu^{norm} == 1)$	Number of normalized Laplacian eigenvalues equal to one.
Number of twos	$\#(\mu^{norm} == 2)$	
Lower slope		Slope of the straight line fitting the normalized Laplacian eigenvalues that fall in the interval $(0, 1)$. The straight line is obtained through the least mean square (LMS) method.
Upper slope		Slope of the straight line fitting the normalized Laplacian eigenvalues that fall in the interval $(1, 2)$. The straight line is obtained through the least mean square (LMS) method.
