# Supplementary material for: Automated Analysis of Proliferating Cells Spatial Organisation Predicts Prognosis in Lung Neuroendocrine Neoplasms
Source: Cancers (Basel). 2021 Sep 29;13(19):4875. doi: 10.3390/cancers13194875 (PMC8508355; doi:10.3390/cancers13194875)
Supplement: Supplementary file 1 [file cancers-13-04875-s001.zip › Supplementary Table S1.pdf]

| Sample ID | Follow-up interval (months) | Prognostic class | N of Ki-67+ cells | Processed tissue area ($mm^2$) | Processed tissue area (*10^8 pixels) | Ki-67+ cells density ($cells/mm^2$) | Ki-67 LI |
|---|---|---|---|---|---|---|---|
| Varese_LCNEC_1 | 5,523287671 | POOR | 19983 | 39,3887676 | 1,911 | 507,3273732 | 60 |
| Turin_AC_1 | 6,21369863 | POOR | 13177 | 220,338004 | 10,69 | 59,80357342 | 10 |
| Nice_LCNEC_1 | 7,528767123 | POOR | 36565 | 49,3029472 | 2,392 | 741,6392341 | 83 |
| Nice_LCNEC_2 | 8,843835616 | POOR | 51164 | 25,1667636 | 1,221 | 2032,998792 | 65 |
| Pisa_LCNEC_1 | 9,369863014 | POOR | 104272 | 69,3992572 | 3,367 | 1502,494468 | 40 |
| Pisa_LCNEC_2 | 11,7369863 | POOR | 138969 | 98,6471176 | 4,786 | 1408,748713 | 75 |
| Nice_LCNEC_3 | 12,2630137 | POOR | 128256 | 65,8952852 | 3,197 | 1946,3608 | 69 |
| Varese_LCNEC_2 | 16,56986301 | POOR | 35339 | 132,8829852 | 6,447 | 265,9407444 | 50 |
| Turin_AC_2 | 19,75890411 | POOR | 74960 | 92,9170928 | 4,508 | 806,7406948 | 15 |
| Pisa_AC_1 | 24,55890411 | POOR | 22079 | 95,7821052 | 4,647 | 230,5127868 | 35 |
| Pisa_LCNEC_3 | 31,79178082 | POOR | 123440 | 43,1194672 | 2,092 | 2862,744093 | 80 |
| Varese_AC_1 | 37,8739726 | POOR | 4935 | 137,685488 | 6,68 | 35,84255735 | 3 |
| Pisa_AC_2 | 38,20273973 | POOR | 51014 | 117,280004 | 5,69 | 434,9761107 | 20 |
| Turin_AC_3 | 47,86849315 | POOR | 24848 | 72,862006 | 3,535 | 341,0282171 | 35 |
| Pisa_AC_3 | 49,24931507 | GOOD | 37723 | 142,0345356 | 6,891 | 265,5903358 | 20 |
| Bari_AC_1 | 49,51232877 | GOOD | 2999 | 146,136244 | 7,09 | 20,52194526 | 2 |
| Turin_AC_4 | 50,95890411 | GOOD | 11283 | 134,3464088 | 6,518 | 83,98438113 | 3 |
| Pisa_AC_4 | 54,57534247 | GOOD | 8103 | 155,2671828 | 7,533 | 52,1874607 | 7 |
| Pisa_LCNEC_4 | 57,20547945 | GOOD | 45445 | 49,2823356 | 2,391 | 922,135679 | 70 |
| Pisa_AC_5 | 58,22465753 | GOOD | 25786 | 100,3166572 | 4,867 | 257,0460452 | 7 |
| Nice_AC_1 | 61,15068493 | GOOD | 31953 | 59,361408 | 2,88 | 538,2790112 | 12 |
| Pisa_AC_6 | 65,26027397 | GOOD | 3340 | 39,574272 | 1,92 | 84,39826764 | 5 |

| | | | | | | | |
|---|---|---|---|---|---|---|---|
| Brussels_LCNEC_1 | 77,12876712 | GOOD | 149392 | 91,8040664 | 4,454 | 1627,291751 | 50 |
| Varese_AC_2 | 78,04931507 | GOOD | 36921 | 181,5469728 | 8,808 | 203,3688551 | 2,5 |
| Pisa_AC_7 | 83,50684932 | GOOD | 16588 | 71,8520376 | 3,486 | 230,8633207 | 5 |
| Pisa_AC_8 | 91,36438356 | GOOD | 33907 | 68,7809092 | 3,337 | 492,9710932 | 15 |
| Nice_LCNEC_4 | 96,65753425 | GOOD | 117984 | 110,5400108 | 5,363 | 1067,342034 | 76 |
| Pisa_LCNEC_5 | 97,15068493 | GOOD | 131912 | 88,5474336 | 4,296 | 1489,732617 | 65 |
| Nice_LCNEC_5 | 108,5917808 | GOOD | 68616 | 86,1358764 | 4,179 | 796,6018675 | 39 |
| Turin_AC_5 | 111,8136986 | GOOD | 3145 | 38,6673616 | 1,876 | 81,33474511 | 4 |
| Nice_LCNEC_6 | 131,5726027 | GOOD | 86812 | 27,2691468 | 1,323 | 3183,524613 | 44 |
