# Supplementary material for: Automated Analysis of Proliferating Cells Spatial Organisation Predicts Prognosis in Lung Neuroendocrine Neoplasms
Source: Cancers (Basel). 2021 Sep 29;13(19):4875. doi: 10.3390/cancers13194875 (PMC8508355; doi:10.3390/cancers13194875)
Supplement: Supplementary file 1 [file cancers-13-04875-s001.zip › Supplementary Table S2.pdf]

Feature name	Category
Hausdorff fractal dimension	Fractality
Higuchi fractal dimension horizontal	"
Higuchi fractal dimension vertical	"
Average Higuchi fractal dimension	"
Shannon entropy (box size 20µm)	Information theory
Shannon entropy (box size 40µm)	"
Shannon entropy (box size 80µm)	"
Shannon entropy (box size 160µm)	"
Shannon entropy (box size 320µm)	"
Shannon entropy (box size 640µm)	"
Number of nodes	Graph theory
25µm - Number of edges	Graph theory (25 µm)
**25µm - Degree**	"
25µm - Percentage of unconnected nodes	"
25µm - Percentage of end nodes	"
25µm - Size of largest connected component normalized	"
25µm - Number of connected components normalized	"
25µm - Average number of nodes in a connected component normalized	"
25µm - Average shortest path length between 2 nodes	"
25µm - Max shortest path length between 2 nodes	"
25µm - Percentage of unconnected pairs of nodes	"
**25µm - Closeness centrality**	"
**25µm - Weighted closeness centrality**	"
**25µm - Betweenness centrality**	"
**25µm - Pagerank centrality**	"
**25µm - Eigenvector centrality**	"
25µm - Global efficiency	"
**25µm - Local efficiency (closed)**	"
**25µm - Local efficiency (open)**	"
**25µm - Clustering coefficient (closed)**	"
**25µm - Clustering coefficient (open)**	"
25µm - Largest eigenvalue (A)	"
25µm - Second largest eigenvalue (A)	"

25µm - Spectral gap	"
25µm - Graph energy	"
25µm - Laplacian energy	"
25µm - Triangles number (A)	"
25µm - Number of ones (L_norm)	"
25µm - Number of twos (L_norm)	"
25µm - Lower slope (L_norm)	"
25µm - Upper slope (L_norm)	"
50µm - Number of edges	Graph theory (50 µm)
**50µm - Degree**	"
50µm - Percentage of unconnected nodes	"
50µm - Percentage of end nodes	"
50µm - Size of largest connected component normalized	"
50µm - Number of connected components normalized	"
50µm - Average number of nodes in a connected component normalized	"
50µm - Average shortest path length between 2 nodes	"
50µm - Max shortest path length between 2 nodes	"
50µm - Percentage of unconnected pairs of nodes	"
**50µm - Closeness centrality**	"
**50µm - Weighted closeness centrality**	"
**50µm - Betweenness centrality**	"
**50µm - Pagerank centrality**	"
50µm - Global efficiency	"
**50µm - Local efficiency (closed)**	"
**50µm - Local efficiency (open)**	"
**50µm - Clustering coefficient (closed)**	"
**50µm - Clustering coefficient (open)**	"
75µm - Number of edges	Graph theory (75 µm)
**75µm - Degree**	"
75µm - Percentage of unconnected nodes	"
75µm - Percentage of end nodes	"
75µm - Size of largest connected component normalized	"
75µm - Number of connected components normalized	"
75µm - Average number of nodes in a connected component normalized	"

**75µm - Closeness centrality**	"
**75µm - Weighted closeness centrality**	"
**75µm - Betweenness centrality**	"
**75µm - Pagerank centrality**	"
**75µm - Eigenvector centrality**	"
**75µm - Local efficiency (closed)**	"
**75µm - Local efficiency (open)**	"
**75µm - Clustering coefficient (closed)**	"
**75µm - Clustering coefficient (open)**	"
Intensity per squared mm	Stochastic geometry
K function (homogeneous) (radius = 5 µm)	"
K function (homogeneous) (radius = 7.5 µm)	"
K function (homogeneous) (radius = 10 µm)	"
K function (homogeneous) (radius = 12.5 µm)	"
K function (homogeneous) (radius = 15 µm)	"
K function (homogeneous) (radius = 17.5 µm)	"
K function (homogeneous) (radius = 20 µm)	"
K function (homogeneous) (radius = 22.5 µm)	"
K function (homogeneous) (radius = 25 µm)	"
K function (homogeneous) (radius = 27.5 µm)	"
K function (homogeneous) (radius = 30 µm)	"
K function (homogeneous) (radius = 32.5 µm)	"
K function (homogeneous) (radius = 35 µm)	"
K function (homogeneous) (radius = 37.5 µm)	"
K function (homogeneous) (radius = 40 µm)	"
K function (homogeneous) (radius = 42.5 µm)	"
K function (homogeneous) (radius = 45 µm)	"
K function (homogeneous) (radius = 47.5 µm)	"
K function (homogeneous) (radius = 50 µm)	"
L function (homogeneous) (radius = 5 µm)	"
L function (homogeneous) (radius = 7.5 µm)	"
L function (homogeneous) (radius = 10 µm)	"
L function (homogeneous) (radius = 12.5 µm)	"
L function (homogeneous) (radius = 15 µm)	"

L function (homogeneous) (radius = 17.5 µm)	"
L function (homogeneous) (radius = 20 µm)	"
L function (homogeneous) (radius = 22.5 µm)	"
L function (homogeneous) (radius = 25 µm)	"
L function (homogeneous) (radius = 27.5 µm)	"
L function (homogeneous) (radius = 30 µm)	"
L function (homogeneous) (radius = 32.5 µm)	"
L function (homogeneous) (radius = 35 µm)	"
L function (homogeneous) (radius = 37.5 µm)	"
L function (homogeneous) (radius = 40 µm)	"
L function (homogeneous) (radius = 42.5 µm)	"
L function (homogeneous) (radius = 45 µm)	"
L function (homogeneous) (radius = 47.5 µm)	"
L function (homogeneous) (radius = 50 µm)	"

<u>statistics on local features</u>

Arithmetic mean
Geometric mean*
Harmonic mean*
Standard deviation
Skewness
Kurtosis
Range
Mode
Minimum
Quartile 1
Median
Quartile 3
Maximum
Second central moment*
Third central moment*
Fourth central moment*

* not computed for Local Efficiencies and Clustering Coefficients
